# MicroRNAs in Takotsubo Syndrome: A Systematic Review of Regulatory Networks in Stress-Induced Cardiomyopathy

**DOI:** 10.3390/ijms26199790

**Published:** 2025-10-08

**Authors:** Domingos Sousa, Filipa Abreu Martins, Ângelo Luís, Pedro Serralheiro

**Affiliations:** 1 RISE-Health, Department of Medical Sciences, Faculty of Health Sciences, University of Beira Interior, Av. Infante D. Henrique, 6200-506 Covilhã, Portugal; fmgam@hotmail.com (F.A.M.); afluis27@gmail.com (Â.L.);; 2 Group of Systematic Reviews of University of Beira Interior (GRUBI), Faculty of Health Sciences, University of Beira Interior, Av. Infante D. Henrique, 6200-506 Covilhã, Portugal; 3 Polytechnic Institute of Viana do Castelo (IPVC), Escola Superior de Saúde, Rua D. Moisés Alves de Pinho, 4900-314 Viana do Castelo, Portugal; 4 Radiotherapy Service, Unidade Local de Saúde São João, Alameda Prof. Hernâni Monteiro, 4200-319 Porto, Portugal; 5 Centro de Investigação em Ciências da Saúde (CICS-UBI), Universidade da Beira Interior, Av. Infante D. Henrique, 6200-506 Covilhã, Portugal; 6 Department of General Surgery, Unidade Local de Saúde de Coimbra, Praceta Prof. Mota Pinto, 3004-561 Coimbra, Portugal; 7 Faculty of Medicine, University of Coimbra, Praceta Prof. Mota Pinto, Azinhaga de Santa Comba, 3000-075 Coimbra, Portugal

**Keywords:** MicroRNAs, Takotsubo Syndrome, stress-induced cardiomyopathy, myocardial remodelling, biomarker, myocardial inflammation, cardiac dysfunction

## Abstract

MicroRNAs (miRNAs) have emerged as crucial regulators of gene expression and have been implicated in various physiological and pathological processes, including cardiovascular diseases. The clinical presentation, diagnostic criteria, and proposed pathophysiological mechanisms of Takotsubo Syndrome (TTS) are discussed, with an emphasis on the emerging evidence implicating miRNAs in its etiology and progression. A systematic review following the PRISMA guidelines was performed on the evidence regarding the interplay between miRNAs and TTS. A search of the Pubmed, Web of Science, and Scopus databases was conducted and resulted in 584 articles. Of these, 14 full-text articles were eligible for inclusion in the qualitative analysis. The reviewed studies suggest that multiple miRNAs are involved in the processes associated with TTS pathophysiology, including acute and chronic myocardial inflammation, oxidative stress, apoptosis, microvascular dysfunction, hypertrophy, and, ultimately, maladaptive cardiac remodelling. This review provides an overview of the current understanding of miRNAs in cardiovascular pathophysiology, with a specific focus on their potential roles in TTS. To the best of our knowledge, this is the first systematic exploration of the miRNAs involved in TTS and its modulation as potential biomarkers or therapeutic targets.

## 1. Introduction

### 1.1. microRNAs

MicroRNAs (miRNAs, MiRs) are a class of single-stranded non-coding RNAs of ~22 nucleotides in length, which negatively regulate gene expression at the post-transcriptional level by binding to a target messenger RNA (mRNA) in a complementary way, and cause gene silencing through the inhibition of translation or by promoting the degradation of mRNA [1].

Over 2600 miRNAs have been studied in human beings in recent years; however, their function is not yet fully understood [2]. miRNAs regulate over 60% of human protein-coding genes [2] and play a crucial role not only in post-transcriptional regulation, but also in the epigenetic modulation of gene expression, mediated by the Argonaute 2 (AGO2) protein [3].

**Figure 1:**
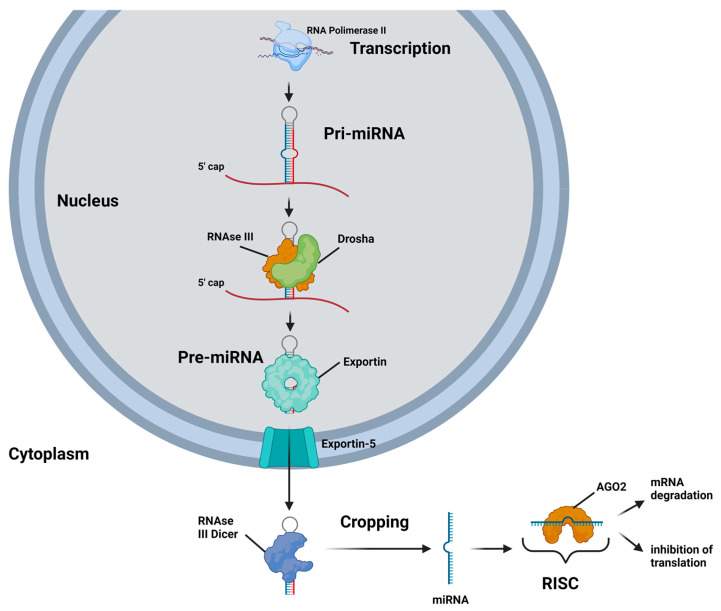
A schematic representation of the biogenesis and mechanisms of action of miRNA is as follows: A miRNA gene is transcribed by RNA polymerase II into a stem-loop primary miRNA (pri-miRNA). Within the nucleus, the pri-miRNA is processed by the RNase III endonuclease Drosha into a hairpin-like precursor miRNA (pre-miRNA). The pre-miRNA is then transported to the cytoplasm by a GTP-dependent protein transporter, exportin-5. In the cytoplasm, pre-miRNAs are cleaved by the RNase III endonuclease Dicer into approximately 22-nucleotide double-stranded miRNAs. After unwinding, both miRNA strands can be functional; however, usually one strand, termed the guide strand, is incorporated into the RNA-induced silencing complex (RISC). The miRNA-RISC, which contains the Argonaute 2 (AGO2) protein, binds to the 3′ untranslated region (3′UTR) of the target mRNAs and causes gene silencing either by inhibiting the translation and/or by mRNA degradation.

MiRNAs are located in intergenic regions, introns, and polycistronic sites, and can be transcribed either individually or in clusters (Figure 1) [4]. They are initially transcribed by RNA polymerase II as primary miRNAs (pri-miRNAs), which possess a stem-loop structure similar to that of mRNAs, complete with a 5′ cap and a 3′ poly A tail [4]. The pri-miRNAs are then cleaved by the RNase III enzyme Drosha into smaller miRNA precursors, known as pre-miRNAs, which are subsequently exported into the cytoplasm by exportin-5 [5]. In the cytoplasm, the RNase III enzyme Dicer further processes the pre-miRNAs into short mature miRNAs, which then associate with the AGO2 protein to form the RNA-induced silencing complex (RISC) [6].

The primary mechanism by which miRNAs exert their regulatory effects on protein-coding genes involves the RISC, which guides the miRNA to its target through complementary base pairing. Typically, the RISC, with its miRNA 5′ end, interacts with the 3′ untranslated region (3′ UTR) of target mRNAs, leading to post-transcriptional gene silencing through translational inhibition or mRNA degradation [1,7]. However, recent research indicates that miRNAs may also engage with the 5′ untranslated region (5′ UTR) or even the coding region of target mRNAs, potentially resulting in translational repression or activation of the target proteins [8,9].

Through gene expression regulation, miRNAs have an important role in various cellular processes such as cell proliferation, differentiation, apoptosis, and stress response. In the cardiovascular system, miRNAs control the development and maintenance of cardiac rhythm, cardiomyocyte growth and contractility, lipid metabolism, plaque formation, and angiogenesis [10].

Notably, one miRNA can regulate multiple mRNAs, while a single mRNA can also be controlled by several miRNAs, highlighting the complexity and versatility of miRNA-mediated gene regulation, thereby playing a crucial role in a variety of physiological and pathological processes [3,7,11]. These properties make them attractive candidates as biomarkers and therapeutic targets in cardiovascular diseases.

### 1.2. Takotsubo Syndrome

Takotsubo Syndrome (TTS) is an acute cardiac syndrome characterized by transient regional wall-motion abnormalities and reversible severe left ventricular dysfunction, which typically recovers spontaneously within hours or weeks. The name of the disease originates from the resemblance of the left ventricle’s shape to a traditional Japanese fishing pot used to trap octopuses [12,13]. TTS typically causes the rapid onset of chest pain, dyspnea, and electrocardiogram changes, often triggered by physical and/or emotional stress, leading to activation of the sympathetic nervous system, myocardial stunning, and left ventricular dysfunction [12].

TTS closely resembles acute coronary syndrome upon presentation, and is, therefore, clinically indistinguishable when presenting with classical symptoms [12,14]. TTS was historically underdiagnosed, but is now recognized as a clinical entity, with an estimated prevalence of 1–3% among patients presenting with suspected ST-elevation myocardial infarction (STEMI) [12,13], with its prevalence increasing to 5–6% among female patients [15]. The recurrence rate of TTS is estimated at 1.8% per patient year [16].

Most TTS patients are women, comprising up to 90% of cases, with an average age range of 65–70 years. Approximately 80% of TTS patients are older than 50 years [17]. However, with increased diagnostic awareness, there has been a rising trend in TTS diagnoses in male patients, particularly following physical stress events [18]. Although more prevalent in older individuals, TTS is not limited to adult patients, and it has been observed in children, including premature neonates [19,20].

While traditionally regarded as a benign condition, recent observational studies have reported rates of cardiogenic shock and mortality comparable to those observed in patients with acute coronary syndrome, which is managed according to current guidelines [21,22]. Upon hospitalization, during the acute phase, hemodynamic and electrical instability can develop in up to one-fifth of TTS patients, leading to complications such as acute heart failure, left ventricular outflow tract obstruction, mitral regurgitation, or cardiogenic shock [21,23]. An analysis of 1750 TTS cases reported a 30-day mortality rate of 5.9%, with a long-term mortality rate of 5.6% per patient per year [16].

Several diagnostic criteria have been proposed (Table S1), including those developed by the Mayo Clinic [24], the Heart Failure Association [25], and the International Takotsubo Registry (InterTAK Registry) [12]. While these criteria share a common framework, the InterTAK criteria uniquely acknowledges the potential coexistence of significant coronary artery disease with TTS and recognizes pheochromocytoma as a potential trigger. To improve diagnostic accuracy and differentiate TTS from acute myocardial infarction (AMI), the InterTAK Diagnostic Score [26] was introduced by the International Takotsubo Registry. This user-friendly scoring system can be quickly calculated at bedside, facilitating timely diagnosis in emergency settings [26].

Although researchers have looked for a genetic predisposition to Takotsubo Syndrome, the results have been inconsistent, and no reproducible variants have been found to explain why it occurs or recurs [21]. This is perhaps not surprising, since TTS is usually triggered by sudden emotional or physical stress rather than by fixed hereditary mutations. MicroRNAs (miRNAs), on the other hand, act as rapid-response regulators of gene expression. They can switch on or off whole networks of genes involved in inflammation, oxidative stress, apoptosis, vascular tone, and energy metabolism—all key processes in TTS. Because they also circulate stably in the blood and can be measured non-invasively, miRNAs provide a window into the body’s immediate molecular reaction to stress. Unlike genetics, which offers only a static picture of risk, miRNAs capture the real-time biology of the syndrome, making them uniquely valuable for understanding TTS and for developing future biomarkers or therapies.

## 2. Unravelling the Enigma of Takotsubo Syndrome: Insights into Pathogenesis and Molecular Pathways from Contemporary Research

The current literature provides evidence for the molecular mechanisms and pathways involved in TTS pathogenesis, which are briefly outlined here.

### 2.1. Sympathetic Stimulation: The Role of Catecholamines

While the precise pathophysiological mechanism behind TTS remains unclear, there is widespread acknowledgment of the crucial role of sympathetic hyperactivity [12]. TTS episodes are often triggered by unexpected emotional or physical stressful situations [27]. In response to stress, the cognitive centres of the brain activate the hypothalamic–pituitary–adrenal (HPA) axis, leading to an increased release of (nor)epinephrine into the bloodstream, causing a sympathetic stimulation of the heart tissue [28]. Interestingly, a recent meta-analysis with 108 patients described only a mild-to-moderate increase in serum catecholamines levels. Another observation is that a significant portion of catecholamines is conveyed directly to myocardial adrenoreceptors, placed at the coronary sinus, by sympathetic nerve endings [29].

Furthermore, iodine-123 meta-iodo-benzyl-guanidine myocardial scintigraphy has shown an acute reduction in cardiac sympathetic neuronal uptake in the affected segments, contrasting with normal perfusion [30,31].

While heightened sympathetic stimulation is fundamental to TTS, the mechanism through which excessive catecholamine levels induce myocardial stunning in the diverse array of regional ballooning patterns; therefore, the characteristics of this syndrome remains unknown. Several mechanisms have been associated with its pathophysiology, but there is no evidence available that determines whether they are a consequence or a concomitant process of the catecholamine surge.

#### 2.1.1. Microvascular Dysfunction

Catecholamines and endothelin predominantly exert their vasoconstrictive effects in the coronary microvasculature, where α-1 receptors and endothelin receptor type A are prevalent [12]. Recent studies have revealed that adenosine intravenous administration leads to a transient improvement in myocardial perfusion and contractility index, suggesting that intense microvascular constriction may play a critical role in the pathogenesis of TTS [32]. Adenosine-mediated vasodilation in smooth muscle and vascular cells serves to counterbalance the pronounced catecholamine-induced vasoconstriction, likely through the activation of Gs protein [33].

Furthermore, the concept that acute microcirculatory dysfunction in TTS is a contributing pathophysiological factor secondary to increased sympathetic stimulation is corroborated by endomyocardial biopsies showing microvascular endothelial cells apoptosis [34]. This microcirculatory dysfunction is known to be transient, but its severity could be a predictor of poor outcomes [35].

#### 2.1.2. Myocardial Inflammation

Myocardial inflammation has been observed during the acute phase, with evidence suggesting its persistence into the subacute and chronic phases, thereby contributing to long-term cardiac dysfunction and symptoms. Cardiac magnetic resonance imaging has been employed to illustrate edema in the effected segments during the acute phase, with some patients showing low-grade features in the chronic phase [36,37]. High concentrations of catecholamines can induce toxic effects on cardiomyocytes, leading to a mononuclear inflammatory response. Endomyocardial biopsies conducted on TTS patients have unveiled contraction band necrosis, a pathological hallmark commonly associated with TTS, which has also been identified in cases of pheochromocytoma and subarachnoid hemorrhage [37,38,39,40]. The excessive catecholamines may trigger oxidative stress and mediate an inflammatory reaction, resulting in the release of interleukins [41]. This mechanism could also be associated with the activation of myocardial survival pathways, directly inhibiting apoptotic mechanisms and cellular function to preserve cell functionality in the context of myocardial stunning [42].

Furthermore, elevated norepinephrine levels correlate with increased C-reactive protein levels and leukocyte counts in acute TTS, suggesting that catecholamines may instigate a systemic inflammatory response [43]. Additionally, studies by Nef et al. [44] have demonstrated significant macrophage infiltration within the myocardium of individuals with TTS. Macrophages contain phagocytic NADPH oxidase (NOX-2), which plays a crucial role in controlling oxidative stress in both myocardial and vascular (endothelial) cells.

Moreover, cases of TTS have been reported following influenza vaccinations and cancer treatments involving immune checkpoint inhibitors, which activate T lymphocytes. This indicates that immune activation and inflammation may serve as triggers for TTS [45]. Whether inflammation acts as a causal trigger in Takotsubo pathogenesis or arises secondarily from catecholamine-induced myocardial damage remains an open and actively debated question.

##### Maladaptive Myocardial Remodelling

Recent evidence reveals that TTS may lead to persistent myocardial alterations well beyond the acute recovery phase [14,37]. Myocardial remodelling, encompassing changes in structure, function, and cellular metabolism, is a key concept in chronic cardiac pathophysiology. In TTS, emerging data suggest that, even after the normalization of left ventricular ejection fraction, patients may experience persistent subclinical myocardial abnormalities. These include impaired myocardial deformation (e.g., reduced longitudinal and circumferential strain), prolonged myocardial edema, increased native T1 values on cardiac magnetic resonance imaging, and energetic deficits demonstrated by reduced phosphocreatine-to-ATP ratios [37]. These maladaptive remodelling processes can translate into functional limitations such as fatigue, dyspnoea, reduced exercise tolerance, and persistent symptoms that meet the criteria for a chronic heart failure phenotype, despite seemingly normal cardiac output at rest [37]. Advanced cardiac imaging and cardiopulmonary exercise testing have been pivotal in revealing these latent abnormalities. Longitudinal studies and cross-sectional comparisons with matched controls have shown that abnormalities in strain imaging, metabolic markers, and myocardial tissue characterization may persist for months to years after an acute episode [14,37]. Given the growing body of evidence suggesting that TTS is not a transient and benign condition but rather one showing potential for the development of maladaptive myocardial remodelling and functional impairment, there is a critical need to consolidate current knowledge.

#### 2.1.3. Metabolic Pathway Dysregulation

In addition to catecholamine toxicity, TTS is characterized by profound metabolic remodelling. Normally, the heart flexibly switches between fatty acid (FA) and glucose oxidation to meet energy demands. In TTS, this adaptability is lost: impaired FA uptake and oxidation lead to toxic lipid accumulation, while glucose utilization is reduced despite preserved perfusion, reflecting catecholamine-induced insulin resistance and flow–metabolism mismatch. Positron emission tomography imaging confirms persistent perfusion–metabolism abnormalities, even after functional recovery [46,47].

Mitochondrial dysfunction further compromises ATP production and increases reactive oxygen species, aggravating calcium mishandling and apoptosis. Ketone bodies may become an alternative energy source, although whether this is adaptive or maladaptive remains uncertain. Beyond the myocardium, systemic alterations, such as insulin resistance and a catabolic–anabolic imbalance, contribute to long-term functional impairment [47].

These findings support the concept that TTS is not merely transient, but involves sustained energetic deficits. Given that microRNAs regulate lipid metabolism, glucose homeostasis, and mitochondrial function, their dysregulation may represent a key link between sympathetic overstimulation and metabolic remodelling in TTS.

This review aims to critically assess the current evidence of miRNAs involvement in the core pathophysiological mechanisms of TTS. We examined studies exploring circulating or tissue-specific miRNAs in cellular, animal, and human models to identify those with the greatest potential for diagnostic, prognostic, or therapeutic application. To our knowledge, no systematic reviews have yet been published specifically addressing the intersection of microRNAs and acute and chronic myocardial inflammation, oxidative stress, apoptosis, microvascular dysfunction, hypertrophy, and, ultimately, maladaptive cardiac remodelling in TTS; therefore, this review seeks to fill that gap and provides a foundation for future translational research in this emerging field.

## 3. Materials and Methods

This work was conducted according to the Preferred Reporting Items for Systematic Reviews and Meta-Analyses (PRISMA) [48,49,50]. The review protocol was registered in PROSPERO (CRD420250399396) to ensure methodological rigour and transparency.

A computer search was conducted on the 28th of January 2025 by two independent authors using three major databases (Pubmed, Scopus, Web of Science) using the following equation, (MicroRNA OR Circulating MicroRNA OR miRNA) AND (Stress Disorder OR Psychological Stress OR Physiologic* Stress OR Metabolic Stress Response OR Biological Stress OR catecholamine OR endothelial dysfunction) AND ((Myocardium AND Inflammation) OR takotsubo OR Contractile dysfunction), and the following search terms: “microRNA OR circulating microRNA” AND “Takotsubo syndrome” or “Stress cardiomyopathy” or “Broken heart syndrome” or “Apical ballooning syndrome” or “Transient left ventricular apical ballooning syndrome” or “Stress-induced cardiomyopathy” or “Ampulla cardiomyopathy” or “Acute reversible left ventricular dysfunction” or “Neurogenic stunned myocardium”.

**Figure 2:**
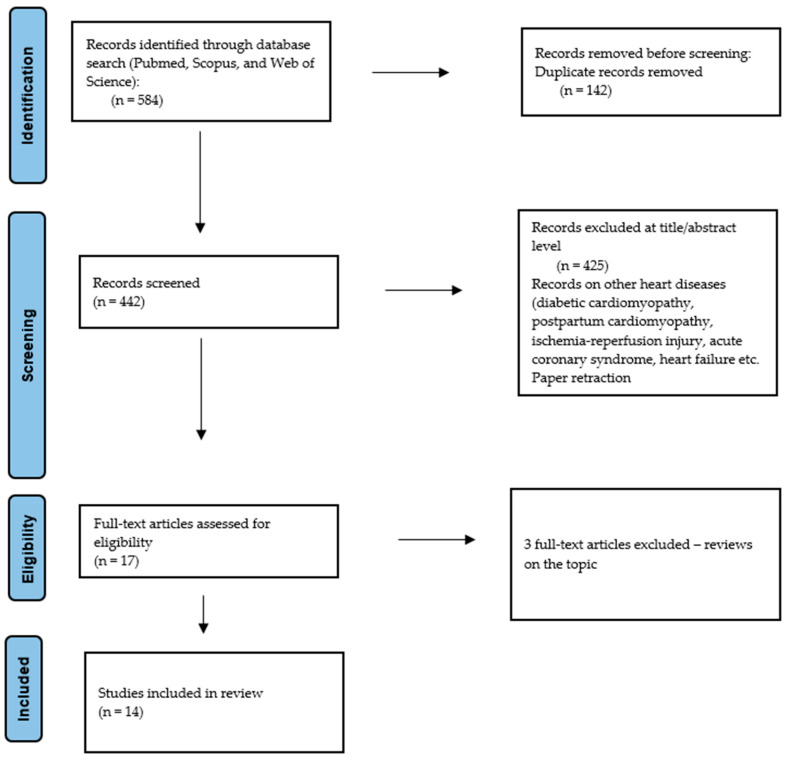
Preferred Reporting Items for Systematic Review (PRISMA) flow chart—search strategy.

After database research, the obtained abstracts were screened by the authors, and only the articles connected to the topic of microRNA, Takotsubo Syndrome, and the pathophysiological process possibly implicated in the disease were included. Articles about acute coronary syndromes, heart failure, and diabetic cardiomyopathy were excluded. Two researchers (D.S., F.A.M.) independently reviewed the documents whose title or abstract seemed relevant, and selected those that analyzed miRNAs. The question of the suitability of miRNAs was resolved by consensus among the researchers. Unpublished studies or grey literature were not included. Data extraction was performed by two researchers (D.S. and F.A.M). Only Portuguese- or English-language papers or abstracts were included in the search. A summary of the methodology applicated on the database research, according to the PRISMA statement, is presented in Figure 2 [48].

## 4. Results

The analysis included a total of 14 full-text original research articles (Figure 2). A complete list of relevant articles on this topic can be found in Table 1. Below, we examine the current evidence in detail.

## 5. Discussion

**Figure 3:**
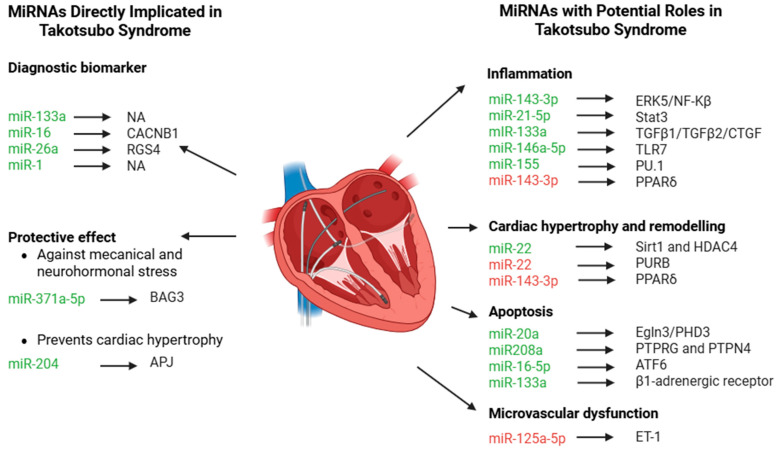
MiRNAs and relative targets associated with Takotsubo Syndrome (TTS) or miRNAs with indirect role in TTS. Green miRNAs are upregulated, while red miRNAs are downregulated in the different pathophysiological mechanisms.

This systematic review highlights the significant potential of miRNAs as key elements in clarifying the pathophysiology of Takotsubo Syndrome (TTS). To improve clarity, this discussion is organized into two sections: (i) miRNAs already known to be directly implicated in TTS, and (ii) those with only indirect or potential effects in TTS (Figure 3).

### 5.1. miRNAs Directly Implicated in Takotsubo Syndrome

#### 5.1.1. miRNAs as Diagnostic Biomarkers in Takotsubo Syndrome

In recent years, miRNAs have gained considerable attention as potential circulating biomarkers due to their stability in plasma and the ability to quantify them efficiently using techniques such as real-time PCR and microarrays [65].

Kuwabara et al. [61] were the first to describe miR-133a as a sensitive biomarker of cardiomyocyte injury and its release into circulation, particularly via exosomes, correlated with the onset of myocardial damage in the TTS subgroup. Subsequently, consistent evidence supports the differential expression of miR-16 and miR-26a in patients with TTS compared to those with AMI or healthy individuals [54]. Jaguszewski et al. [54] demonstrated that a panel of circulating miRNAs—miR-16, miR-26a, miR-1, and miR-133a—could robustly distinguish TTS from STEMI, achieving an area under the curve (AUC) of 0.881, with 96.77% sensitivity and 70.37% specificity.

Importantly, miR-16 and miR-26a, known for their involvement in stress and depression pathways, were significantly upregulated in TTS, but not in AMI. Their elevation aligns with the well-documented psychosocial triggers of TTS and supports the hypothesis of neuro-cardiogenic mechanisms involving the central nervous system and stress-axis dysregulation. In contrast, miR-1 and miR-133a, both well-established markers of myocardial injury, were found to be more elevated in AMI than in TTS, although still raised in both compared to healthy controls.

Further mechanistic insight is provided by Couch et al., who found that miR-16 and miR-26a are not only elevated in circulation, but also functionally contribute to TTS pathogenesis [55]. In both rodent and human cardiomyocyte models, these miRNAs increased susceptibility to adrenaline-induced apical dysfunction, reproducing the hallmark contractile abnormalities of TTS. Mechanistically, luciferase assays demonstrated that miR-16 reduced expression of CACNB1 (L-type calcium channel Cavβ subunit), while miR-26a suppressed RGS4 (regulator of G-protein signalling 4)—both of which are essential for normal myocardial contraction and the prevention of conduction abnormalities.

Notably, Couch et al. also showed that adrenaline administration alone did not stimulate miR-16 or miR-26a expression, indicating that their upregulation is not a direct consequence of acute catecholamine exposure [55]. Instead, this suggests a baseline molecular predisposition, potentially shaped by chronic psychological stress or underlying neuropsychiatric conditions. Since these miRNAs are elevated in stress, anxiety, and depression, they may form part of a priming mechanism wherein chronic emotional or psychological stress—a known risk factor in TTS patients—predisposes the heart to acute injury upon subsequent catecholamine surges.

Taken together, these findings support the potential of miR-16 and miR-26a not only as diagnostic biomarkers, but also as mediators of TTS pathogenesis.

#### 5.1.2. miRNAs with Potential Protective Effect

One of the proteins responsible for maintaining cardiomyocyte homeostasis is BAG3 (Bcl-2-associated athanogene 3). BAG3 plays a critical role in cardiomyocytes by supporting sarcomeric assembly, maintaining cytoskeletal integrity, and facilitating chaperone-assisted autophagy. It is essential for protecting myocardium against mechanical and neurohormonal stress. Recent studies by D’Avenia et al. [52] in patients with TTS identified a novel signalling pathway in which miR-371a-5p modulates BAG3 expression. The authors demonstrated that epinephrine stimulation leads to increased expression of miR-371a-5p, which in turn enhances BAG3 protein levels, likely through translational regulation rather than changes in mRNA stability. This mechanism appears to function as a physiological adaptive response, strengthening cardiomyocyte resilience under acute stress. Importantly, the study also identified a g2252c mutation in the 3′ untranslated region (3′UTR) of the BAG3 gene, which reduces miR-371a-5p binding. This mutation impairs the upregulation of BAG3 in response to stress, leading to a blunted protective response to catecholaminergic stimulation. As a result, myocardial susceptibility to epinephrine-induced injury may be increased. The inability to sufficiently induce BAG3 expression may compromise sarcomere stability and contractile support, thereby contributing to the development of cardiomyocyte damage in TTS.

In parallel, Gaddam et al. [53] provided important information regarding miR-204 function. Their studies found out that miR-204 regulates segmental myocardial motion, particularly under catecholamine-induced stress, a central trigger in TTS. Although its absence did not exaggerate TTS-like dysfunction, it modulated the pattern and timing of segmental contraction: the peak cardiac muscle motion time in the base of the left ventricle was significantly earlier in the miR-204^−/−^ mice. These findings suggest that miR-204 influences regional contractility under adrenergic stress.

Additional work by the same group provides compelling evidence that miR-204 plays a protective and regulatory role in the heart’s response to mechanical and neurohormonal stress [66]. The researchers demonstrated that miR-204 is upregulated in cardiomyocytes during cardiac stress and that, functionally, miR-204 helps prevent cardiac hypertrophy and dysfunction by modulating the behaviour of the apelin receptor (APJ), a G-protein-coupled receptor involved in cardiovascular signalling. MiR-204 inhibits the ability of APJ to mediate maladaptive signalling by promoting its endocytosis via an alternative route, which contributes to the cardioprotective effects of miR-204. Interestingly, the authors also stained cardiac sections of miR-204^−/−^ mice revealing enhanced fibrosis, thereby demonstrating the in vivo cardioprotective role of miR-204 [66].

Collectively, these findings suggest that both miR-371a-5p and miR-204 participate in protective regulatory pathways that may mitigate the effects of acute stress on the heart. Disruption of these mechanisms—through genetic variants or altered expression—could contribute to TTS pathophysiology and may offer targets for future diagnostic or therapeutic strategies.

#### 5.1.3. Microvascular Dysfunction

Microvascular dysfunction has been proposed as a contributing factor in the pathogenesis of TTS, particularly given the frequent absence of significant epicardial coronary artery disease in affected patients [12,14,45]. Impairments in coronary microcirculation may exacerbate myocardial stress and contribute to the regional wall motion abnormalities observed during acute episodes [67].

Endothelin-1 (ET-1), a potent vasoconstrictor—approximately 100 times more powerful than norepinephrine—has been implicated in the microvascular spasm hypothesis of TTS. Jaguszewski et al. identified a significant downregulation of miR-125a-5p in TTS patients, accompanied by a marked increase in plasma ET-1 levels [54]. Given that miR-125a-5p is known to target the 3′-UTR of prepro-ET-1 mRNA, its downregulation could contribute to increased ET-1 expression, potentially promoting exaggerated vasoconstriction and impaired coronary microvascular perfusion. Notably, elevated ET-1 levels have also been associated with microvascular obstruction and a reduced myocardial salvage index following myocardial infarction—effects that underscore its broader pathological role across various acute and chronic cardiovascular conditions [68].

While direct evidence linking miR-125a-5p to TTS remains limited, its well-established role in endothelial regulation supports its plausible contribution to the coronary microvascular disturbances observed in this syndrome.

### 5.2. miRNAs with Indirect or Potential Roles in TTS

#### 5.2.1. Inflammation

An increasing body of evidence indicates that myocardial inflammation occurs during the acute phase of TTS. If persistent into the subacute and chronic phases, this inflammation may play a significant role in the development of long-term cardiac dysfunction and clinical symptoms [45].

MiR-143-3p appears to play a pro-inflammatory role in the context of myocardial hypertrophy. Its upregulation has been associated with activation of the ERK5/NF-κB signalling axis, while its inhibition attenuates the expression of pro-inflammatory markers and enhances the expression of anti-inflammatory mediators such as PPARδ. These effects suggest that miR-143-3p contributes to maladaptive cardiac remodelling by amplifying inflammatory responses that promote myocardial hypertrophy [58].

In contrast, miR-21-5p has emerged as a potential biomarker of inflammation-related cardiac injury. Its expression was shown to localize predominantly within inflammatory cell infiltrates in injured myocardial tissue, and its regulatory relationship with Stat3 further supports its involvement in inflammatory activation. This dual role—as both a marker and a modulator—highlights the relevance of miR-21-5p in the immune landscape of cardiac injury, and possibly in TTS [59].

Additionally, miR-133a has been associated with beneficial effects in inflammatory cardiomyopathy (iCMP). Higher myocardial levels of this miRNA are associated with reduced immune cell infiltration, decreased fibrosis, preserved left ventricular function at the initial diagnosis of iCMP, and showed improvements in left ventricular function at a 12-month follow-up [56]. MiR-133a has been shown to regulate myocardial collagen production by repressing transforming growth factor (TGF)-*β*1 and TGF-*β* and connective tissue growth factor (CTGF) in in vitro models [69]. Also, the authors further revealed an association between the increase levels of miR-155 and inflammatory cell count in endomyocardial biopsies during the acute phase of iCMP. From a pathophysiological standpoint, miR-155 has been suggested to act, at least in part, via targeting the expression of PU.1, an inhibitor of dendritic cell antigen presentation to T-lymphocytes. These miR-155 seems to inhibit dendritic cells presentation to T-lymphocytes. However, miR-155 did not improve the left ventricular function either acutely or 12 months later.

The innate immune system can be activated by miR-146a-5p through Toll-like receptor 7 (TLR7)-dependent signalling, leading to cytokine production and the recruitment of immune cells. Extracellular vesicles carrying miR-146a-5p activate an inflammatory response that affects cardiomyocytes, coronary artery endothelial cells, and cardiac fibroblasts, causing impaired endothelial barrier function and, ultimately, reducing cardiomyocyte contractility [57].

Taken together, the evidence indicates that distinct miRNAs may act as either pro- or anti-inflammatory mediators in the myocardium. Interestingly, it is modulation can have impact on the chronic inflammatory process of TTS and subsequent maladaptive cardiac remodelling and impaired cardiac function [14,37].

#### 5.2.2. Apoptosis

Apoptotic signalling plays a central role in the myocardial dysfunction observed in TTS. In the context of acute cardiac stress, multiple downstream effects—such as oxidative stress, mitochondrial injury, and intracellular calcium overload—converge to activate apoptotic pathways. Several miRNAs have been identified as modulators of these cellular responses in cardiomyocytes, offering mechanistic insights into TTS.

Castaldi et al. demonstrated that overexpression of miR-133a attenuates apoptosis and reduces fibrosis by modulating the β1-adrenergic receptor transduction cascade, thereby limiting maladaptive sympathetic signalling [70]. Similarly, miR-20a exerts anti-apoptotic effect in hypoxia/reoxygenation conditions, a common model for myocardial stress. Its upregulation leads to the direct suppression of Egln3/PHD3, a pro-apoptotic factor, thereby reducing caspase-dependent cell death and promoting cell survival under acute stress. This stress-responsive behaviour positions miR-20a as a potential compensatory mechanism in TTS, where acute sympathetic activation and transient ischemia are central features [63].

Unlike some miRNAs that seem to offer protection under stress, miR-208a appears to have the opposite effect [60]. This cardiac-specific miRNA, encoded by the α-myosin heavy chain gene, has been shown to promote apoptosis in response to oxidative stress. In models using H_2_O_2_ exposure, increased levels of miR-208a were associated with higher reactive oxygen species (ROS) production and activation of caspase-3. It also seems to interfere with survival signalling by targeting phosphatases like protein tyrosine phosphatase receptor type 4 (PTPRG) and protein tyrosine phosphatase non-receptor type 4 (PTPN4), which regulate pro-apoptotic cellular functions. Both phosphatases play a role in maintaining the balance of tyrosine phosphorylation, a key signalling mechanism in cell survival. Notably, restoring their expression helped counteract the pro-apoptotic effects of miR-208a, supporting their protective role. These results suggest that blocking miR-208a might help to protect the heart from oxidative damage. By preserving the activity of PTPRG and PTPN4—two proteins involved in regulating stress signalling—an anti-miR-208a strategy could potentially reduce ROS levels and limit myocardial injury. This approach might be especially relevant in TTS, where acute stress and oxidative injury are central features.

Previous studies revealed increased levels of miR-16-5p during the acute phase of TTS [54,55]. More recently, Toro et al. demonstrated that miR-16-5p contributes to oxidative and endoplasmic reticulum (ER) stress-induced injury in human cardiomyocytes by targeting activating transcription factor 6 (ATF6), a key transcription factor involved in the reduction in unfolded proteins within cytoplasm [62]. ER lumen full of unfolded and misfolded proteins induces ER stress has been associated with diverse cardiac diseases in recent years [71]. The increased folding during ER stress induces ROS production and exacerbates oxidative stress. Ultimately, this cascade can trigger apoptosis. Interestingly, Toro et al. identified ATF6, a key transcription factor involved in the unfolded protein response, as a direct target of miR-16-5p, confirmed using dual-luciferase reporter assays. Under normal conditions, ATF6 plays a protective role by helping cells respond to ER stress and restore homeostasis. However, when miR-16-5p is overexpressed, ATF6 is suppressed, leading to heightened ROS production, increased inflammation, and greater susceptibility to apoptosis. Importantly, blocking miR-16-5p restored ATF6 levels, resulting in reduced oxidative damage, lower inflammatory signalling, and improved cardiomyocyte survival. These findings suggest that inhibiting miR-16-5p could be a promising strategy to protect the heart from stress-induced injury.

Taken together, these miRNAs—miR-133a, miR-20a, miR-208a and miR-16-5p—represent critical modulators of cardiomyocyte apoptosis in the context of cardiomyocyte stress. Their roles not only deepen our understanding of the potential molecular mechanisms driving TTS, but also identify putative therapeutic targets for limiting myocardial injury in this syndrome.

#### 5.2.3. Cardiac Hypertrophy and Remodelling

Structural remodelling and hypertrophy are typical cardiac responses to sustained hemodynamic or neurohormonal stress. Persistent or maladaptive remodelling, however, is associated with myocardial dysfunction, arrhythmogenesis, and progression to heart failure [72].

Functionally, miR-22 regulates genes involved in calcium handling, and sarcomere organization of cardiomyocytes. The study by Gurha et al. explored the effects of genetic deletion of miR-22, finding that miR-22^−/−^ mice were vulnerable to stress-induced cardiac dysfunction, particularly under pressure-overload conditions (e.g., transverse aortic constriction) [64]. These mice displayed impaired calcium handling, reduced SERCA2a (sarcoplasmic reticulum Ca2+ ATPase) activity, and suppressed expression of contractile and Z-disc–associated genes. A key mechanistic insight was that miR-22 suppresses the transcriptional repressor PURB (purine-rich element-binding protein B), which otherwise downregulates serum response factor (SRF)-dependent cardiac gene programmes. In the absence of miR-22, PURB is upregulated, contributing to impaired sarcomere gene expression, fibrosis, and maladaptive remodelling. The findings were later confirmed by Huang et al., who demonstrated that miR-22 is upregulated in cardiomyocytes exposed to pressure overload and β-adrenergic stimulation [63]. In a mouse model, chronic infusion of the β-agonist isoproterenol induced ventricular hypertrophy [63]. They extended the study using miR-22 knockout mice. Although these mice were initially protected from developing hypertrophy under pressure overload, they ultimately developed dilated cardiomyopathy. This was linked to an increase in cell death and fibrosis in the heart tissue, which the authors confirmed using histological analysis. In-depth cell pathway studies allowed for the confirmation that miR-22 represses sirtuin 1 (sirt1) and histone deacetylase 4 (HDAC4). Both proteins are essential to the maintenance of regular cellular homeostasis because they increase the resistance against stress-induced damage.

Collectively, these studies highlighted that the prolonged downregulation of miR-22 under stress conditions can lead to abnormal calcium handling, increased apoptosis and fibrosis, and, ultimately, maladaptive cardiac remodelling. These mechanisms may contribute to the pathophysiology of TTS, and they warrant further investigation in both experimental and clinical settings.

## 6. Conclusions

The present systematic review emphasizes the emerging role of circulating microRNAs as potential biomarkers, mechanistic contributors, and therapeutic targets in TTS. Among the investigated miRNAs, miR-371a-5p, miR-204, miR-16-5p, miR-26a, miR-125a-5p, miR-133a, and miR-1 were the ones most consistently associated with TTS. These small non-coding RNAs influence processes ranging from diagnostic differentiation to cardiomyocyte homeostasis, cardiac remodelling, and microvascular stability, thereby emerging as central regulators of stress-induced cardiac dysfunction. In addition, miR-143-3p, miR-21-5p, miR-146a-5p, miR-20, miR-208a, and miR-22 have been studied in cardiac tissue, where they are implicated in inflammation, apoptosis, and cardiac hypertrophy, and thus warrant further investigation in the context of TTS.

## Figures and Tables

**Table 1:** A list of research studies evaluating the role of miRNAs in the mechanisms involved in Takotsubo Syndrome, organized according to the relevant sections.

No.	miRNA	Ref.	Type of Study	Technique ^1^	Main Conclusion ^2^
	miRNAs Implicated in Protective Mechanisms				
1	miR-371a-5p	d’Avenia, M. et al. (2015) [51]	Human and in vivo	qRT-PCR, pMir dual-luciferase assay	Polymorphism of the BAG3 3′UTR reduces miR-371a-5p binding and predisposes cardiomyocytes to epinephrine-induced damage.
2	miR-204	Gaddam, R.R. et al. (2023) [52]	In vivo	qRT-PCR	miR-204^−/−^ mice have early LV anterior heart base movement during phenylephrine exposure.
miRNAs with Diagnostic Relevance					
3	miR-1 miR-16 miR-26a miR-133a miR-125a-5p	Jaguszewski, M. et al. (2014) [53]	Human	RT qRT-PCR	miR-16, miR-26a, miR-1, and miR133a were significantly upregulated in TTS patients compared to healthy controls. miR-125a-5p, which targets ET-1, was downregulated in TTS patients.
4	miR-16 miR-26a	Cough, L. et al. (2022) [54]	Animal	RT qRT-PCR Western blot	miR-16 and miR-26a sensitize the heart to TTS.
	Inflammation				
5	miR-21 miR-126 miR-133a miR-146a miR-155 miR-206	Besler, C. et al. (2016) [55]	Human and in vitro	RT qRT-PCR	Increased endomyocardial miR-133a expression was associated with reduced fibrosis and necrosis, preserved LV systolic function at initial iCMP diagnosis, and improved LV function during follow-up.
6	miR-146a-5p	Shimada, B. et al. (2022) [56]	Animal and in vitro	Extracellular administration of miR-146a-5p mimics	Extracellular miR-146a-5p activates multiple cardiac cells and induces myocardial inflammation and cardiomyocyte dysfunction (reduced CM sarcomere shortening) via intercellular interaction and innate immune TLR7 nucleic acid sensing.
7	miR-143-3p	Yu, B. et al. (2018) [57]	Animal and in vitro	RT qRT-PCR Western blot dual-luciferase assay	The upregulation of miR-143-3p expression increases inflammation in cardiac tissue, consequently leading to cardiac hypertrophy.
8	miR-21-5p	Gryshkova, V. et al. (2018) [58]	In vivo	qRT-PCR in situ hybridization	miR-21-5p is upregulated in inflammatory cardiac processes. miR-21-5p appears to be specific to inflammatory cell infiltrates in the heart.
Apoptosis					
9	miR-208a	Liu, A. et al. (2018) [59]	In vitro	qRT-PCR miR-208a mimic	Anti-miR-208a mitigates ROS-related myocardial injury by reducing oxidative stress and subsequent cellular apoptosis through targeting PTPRG and PTPN4.
10	miR-1 miR-133a	Kuwabara, Y. et al. (2011) [60]	Human and animal	RTqRT-PCR in situ hybridization	Circulating miR-133a serves as a sensitive early biomarker of myocardial injury due to Takotsubo Syndrome.
11	miR-16-5p	Toro, R. et al. (2022) [61]	In vitro	RT-qPCR Western blot	miR-16-5p suppression decreases apoptosis, inflammation, and cardiac damage via activating the ATF6-mediated cytoprotective pathway.
12	miRNA-20a	Frank, D. et al. (2012) [62]	In vitro	RT-qRT-PCR immunoblotting	miR-20a is a cardioprotective microRNA that is rapidly upregulated in cardiomyocytes in response to acute biomechanical stress, thereby inhibiting apoptosis primarily through the targeting and downregulation of proapoptotic factors such as Egln3/PHD3.
Cardiac hypertrophy					
13	miR-22	Huand, Z. et al. (2013) [63]	Animal and in vitro	Northern blot RT-qPCR	miR-22 plays a critical role in cardiomyocyte hypertrophy and cardiac remodelling in response to stress. HDAC4 and *Sirt*1 were identified as targets of miR-22 in the heart.
14	miR-22	Gurha, P. et al. (2012) [64]	In vivo	Western blot qRT-PCR	miR-22 deletion impairs the heart’s response to acute dobutamine stress, disrupting Ca2+ homeostasis and myofibrillar protein regulation. The targeted deletion of microRNA-22 increases the heart’s vulnerability to stress by disrupting protective gene regulation, leading to enhanced cardiac dysfunction and adverse remodelling.

^1^ RT qRT-PCR—Reverse Transcriptase quantitative Real-Time Polymerase Chain Reaction; qRT-PCR—quantitative Real-Time Polymerase Chain Reaction. ^2^ ET-1—endothelin-1; iCMDP—inflammatory cardiomyopathy; LV—left ventricular; iDCM—ischemic dilated cardiomyopathy.

## Data Availability

Data will be available by request to the corresponding author.

## Supplementary Materials

The following supporting information can be downloaded at: https://www.mdpi.com/article/10.3390/ijms26199790/s1.
