# Supplementary material for: MicroRNAs in Takotsubo Syndrome: A Systematic Review of Regulatory Networks in Stress-Induced Cardiomyopathy
Source: Int J Mol Sci. 2025 Oct 8;26(19):9790. doi: 10.3390/ijms26199790 (PMC12525371; doi:10.3390/ijms26199790)
Supplement: Supplementary file 1 [file ijms-26-09790-s001.zip › Flowchart PRISMA 2025_Domingos Sousa.pdf]

PRISMA 2020 flow diagram for new systematic reviews which included searches of databases and registers only

2025

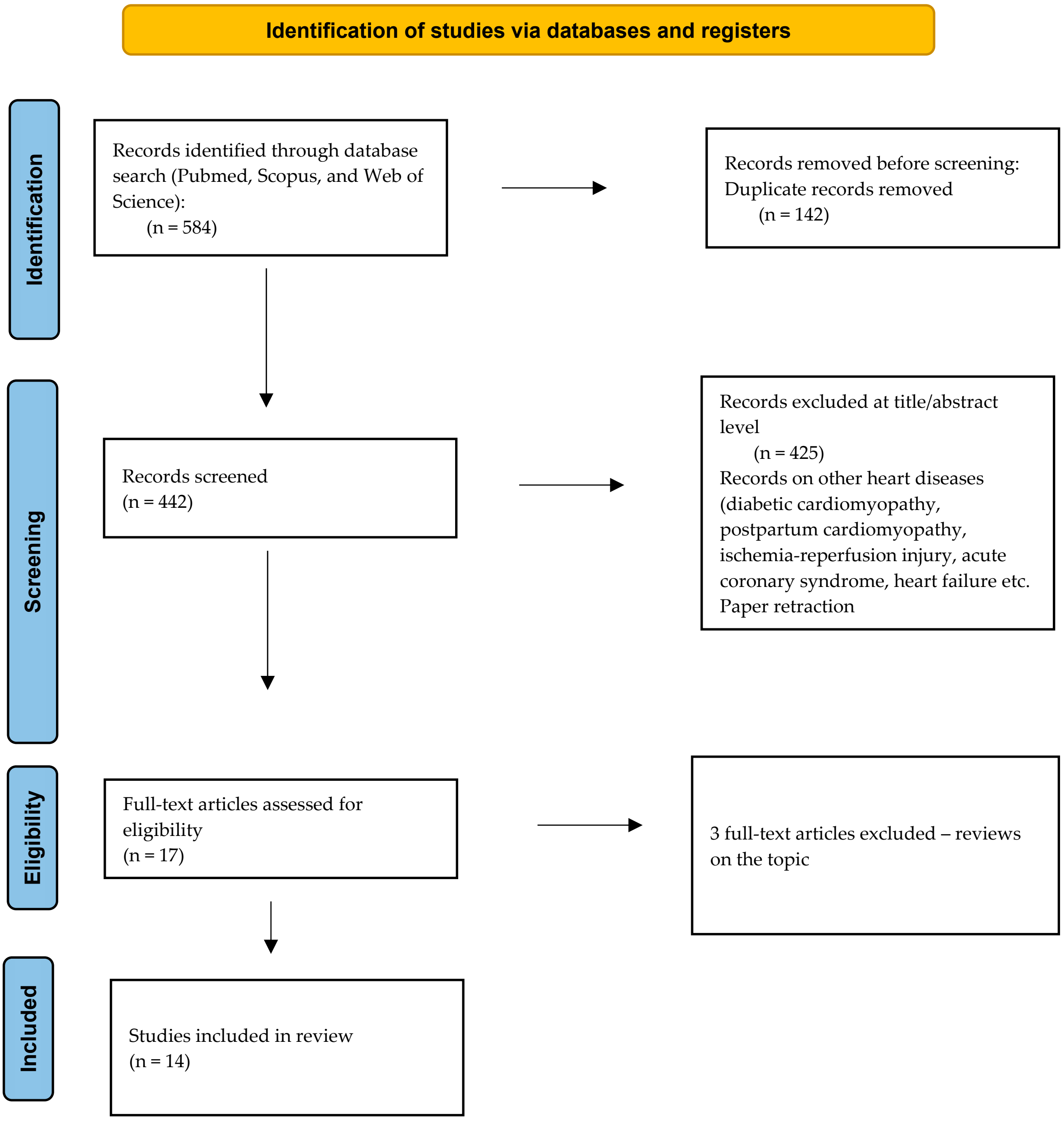

*Consider, if feasible to do so, reporting the number of records identified from each database or register searched (rather than the total number across all databases/registers).

**If automation tools were used, indicate how many records were excluded by a human and how many were excluded by automation tools.

From: Page MJ, McKenzie JE, Bossuyt PM, Boutron I, Hoffmann TC, Mulrow CD, et al. The PRISMA 2020 statement: an updated guideline for reporting systematic reviews. BMJ 2021;372:n71. doi: 10.1136/bmj.n71

For more information, visit: http://www.prisma-statement.org/
