# Supplementary material for: MicroRNAs in Takotsubo Syndrome: A Systematic Review of Regulatory Networks in Stress-Induced Cardiomyopathy
Source: Int J Mol Sci. 2025 Oct 8;26(19):9790. doi: 10.3390/ijms26199790 (PMC12525371; doi:10.3390/ijms26199790)
Supplement: Supplementary file 1 [file ijms-26-09790-s001.zip › Table S1_Domingos Sousa.pdf]

Table S1. Main Takotsubo syndrome diagnostic criteria.

Criteria Set	Diagnostic Criteria
**Revised Mayo Clinic Criteria [24]**	(I) Transient hypokinesis, akinesis, or dyskinesis of the left ventricular mid segments with or without apical involvement; the regional wall motion abnormalities extend beyond a single epicardial vascular distribution; a stressful trigger is often, but not always, present.<br>(II) Absence of obstructive coronary disease or angiographic evidence of acute plaque rupture.<br>(III) New electrocardiographic abnormalities (either ST-segment elevation and/or T-wave inversion) or modest elevation in cardiac troponin.<br>(IV) Absence of pheochromocytoma and myocarditis.
**Heart Failure Association-European Society of Cardiology Criteria [25]**	(I) Transient regional wall motion abnormalities of LV or RV myocardium, which are frequently, but not always, preceded by a stressful trigger (emotional or physical).<br>(II) The regional wall motion abnormalities usually extend beyond a single epicardial vascular distribution and often result in circumferential dysfunction of the ventricular segments involved.<br>(III) Absence of culprit atherosclerotic coronary artery disease, including acute plaque rupture, thrombus formation, and coronary dissection, or other pathological conditions explaining the temporary LV dysfunction (e.g., hypertrophic cardiomyopathy, viral myocarditis).<br>(IV) New and reversible ECG abnormalities (ST-segment elevation, ST-segment depression, LBBB, T-wave inversion, and/or QTc prolongation) during the acute phase (3 months).<br>(V) Significantly elevated serum natriuretic peptide (BNP or NT-proBNP) during the acute phase.<br>(VI) Positive but relatively small elevation in cardiac troponin measured with a conventional assay (disparity between the troponin level and the amount of dysfunctional myocardium present).<br>(VII) Recovery of ventricular systolic function on cardiac imaging at follow-up (3–6 months) .
**International Takotsubo Diagnostic Criteria (InterTAK Registry Diagnostic Criteria) [12]**	(I) Patients show transient left ventricular dysfunction (hypokinesia, akinesia, or dyskinesia) presenting as apical ballooning or midventricular, basal, or focal wall motion abnormalities. Right ventricular involvement can be present. Transitions between all types can exist. The regional wall motion abnormality usually extends beyond a single epicardial vascular distribution; however, rare cases can exist where the abnormality is present in the subtended myocardial territory of a single coronary artery (focal TTS).<br>(II) An emotional, physical, or combined trigger can precede the TTS event, but this is not obligatory.<br>(III) Neurologic disorders (e.g., subarachnoid hemorrhage, stroke/transient ischemic attack, or seizures) as well as pheochromocytoma may serve as triggers for TTS.<br>(IV) New ECG abnormalities are present (ST-segment elevation, ST-segment depression, T-wave inversion, and QTc prolongation); however, rare cases exist without any ECG changes.<br>(V) Levels of cardiac biomarkers (troponin and creatine kinase) are moderately elevated in most cases; significant elevation of brain natriuretic peptide is common.<br>(VI) Significant coronary artery disease is not a contradiction in TTS.<br>(VII) Patients have no evidence of infectious myocarditis.<br>(VIII) Postmenopausal women are predominantly affected.

LV, left ventricle; RV, right ventricle; ECG, electrocardiography; LBBB, left bundle branch block; QTc, QT interval corrected for heart rate; BNP, B-type natriuretic peptide; NT-BNP, N-terminal pro-B-type natriuretic peptide; TTS, Takotsubo syndrome; InterTAK Registry, International Takotsubo Registry; ABS, apical ballooning syndrome.
