# Supplementary material for: MicroRNAs in Takotsubo Syndrome: A Systematic Review of Regulatory Networks in Stress-Induced Cardiomyopathy
Source: Int J Mol Sci. 2025 Oct 8;26(19):9790. doi: 10.3390/ijms26199790 (PMC12525371; doi:10.3390/ijms26199790)
Supplement: Supplementary file 1 [file ijms-26-09790-s001.zip › Searching query.pdf]

Searching query:

(MicroRNA OR Circulating MicroRNA OR miRNA) AND (Stress Disorder OR Psycholog-ical Stress OR Physiologic* Stress OR Metabolic Stress Response OR Biological Stress OR catecholamine OR endothelial dysfunction) AND ((Myocardium AND Inflammation) OR takotsubo OR Contractile dysfunction) and search terms: “microRNA OR circulating mi-croRNA” AND “Takotsubo syndrome”or "Stress cardiomyopathy" or "Broken heart syn-drome" or "Apical ballooning syndrome" or "Transient left ventricular apical ballooning syndrome" or "Stress-induced cardiomyopathy" or "Ampulla cardiomyopathy" or "Acute reversible left ventricular dysfunction" or "Neurogenic stunned myocardium".
